# Chronic Granulomatous Disease: Clinical and Molecular Characterization of Brazilian Patients

**DOI:** 10.1002/jgm.70086

**Published:** 2026-02-06

**Authors:** Leonardo Martinello da Rosa, Martha Braun da Rosa, Mariana de Sampaio Leite Jobim Wilson, Ida Vanessa Doederlein Schwartz, Fernanda Sperb‐Ludwig

**Affiliations:** ^1^ Programa de Pós‐Graduação em Genética e Biologia Molecular (PPGBM) Universidade Federal do Rio Grande do Sul (UFRGS) Porto Alegre Rio Grande do Sul Brazil; ^2^ Hospital de Clínicas de Porto Alegre (HCPA), Centro de Pesquisa Experimental (CPE), Basic Research and Advanced Investigation in Neurosciences Laboratory (BRAIN) Porto Alegre Rio Grande do Sul Brazil; ^3^ Hospital Moinhos de Vento (HMV) Porto Alegre Rio Grande do Sul Brazil; ^4^ InRaras ‐ Brazilian Institute on Rare Diseases Porto Alegre Brazil

**Keywords:** chronic granulomatous disease, genetic diagnosis of immunodeficiencies, inborn errors of immunity, NADPH oxidase, respiratory burst

## Abstract

Chronic granulomatous disease (CGD) is a rare inborn error of immunity caused by defects in components of the NADPH oxidase that impair the elimination of infectious microorganisms. Individuals affected by CGD become more susceptible to recurrent and severe infections. Six male patients from Southern Brazil were clinically and genetically analyzed through data collection from medical records and massively parallel sequencing by a panel for the following genes: *CYBB*, *CYBA*, *NCF1*, *NCF2*, and *NCF4* and whole genome sequencing analysis. The gene‐scan technique was used to identify the GT deletion in *NCF1*. The most common affected organs were the lungs, skin, and lymph nodes; the most common clinical manifestations were recurrent pneumonia, cutaneous involvement, lymph node manifestations, and failure to thrive. Four patients were identified with variants in *CYBB*: p.Cys257Ser, which is novel; p.Cys257Arg; p.Arg157Ter; and p.Trp483Ter. Both missense variants damage the loop E in gp91^phox^, a region with functional and structural relevance for the protein. Functional studies show the expression absence of the protein in patients with the variant p.Arg157Ter. The variant p.Trp483Ter is predicted to undergo nonsense mRNA‐mediated decay. The GT deletion in *NCF1* was identified in two siblings from consanguineous parents: one homozygous and the other apparently heterozygous for the deletion, both with a clinical diagnosis of CGD. Variant analysis in this gene is particularly challenging due to the presence of pseudogenes. A hypothesis for this genotypic discrepancy is the occurrence of a second type of pseudogene lacking the GT deletion, which may have arisen in one parent and been transmitted to the patient observed as heterozygous, being misinterpreted in the analyses as a functional *NCF1* sequence.

## Graphical abstract

Chronic granulomatous disease is a rare inborn error of immunity characterized by the deficient production of reactive oxygen species in phagocytes, which are used in pathogen elimination. Affected individuals become susceptible to severe infections. This study intended to genetically diagnose a cohort of Brazilian patients with suspected of the disease.

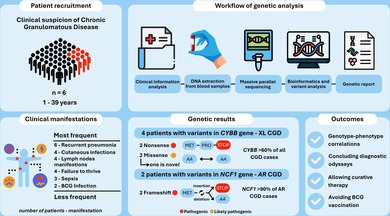

## 1 Introduction

Chronic granulomatous disease (CGD) is a rare inborn error of immunity (IEI), with an overall estimated incidence of 1 in every 250,000 live births, caused by defects in the components of nicotinamide adenine dinucleotide phosphate oxidase complex (NADPH oxidase). The complex is present in phagocytic cells and is responsible for producing reactive oxygen species (ROS), which are essential for the elimination of pathogens. The loss or decrease in ROS production due to defects in the NADPH oxidase compromises its microbicidal function [1, 2]. Affected individuals become more susceptible to bacterial and fungal infections, inflammatory complications, autoimmunity, and malignancies and are commonly infected by a narrow spectrum of pathogens, typically catalase producers. The most common pathogens reported in North America and Europe infecting patients with CGD include *Staphylococcus aureus* and species of the genera *Serratia*, *Nocardia*, *Burkholderia*, *Salmonella*, *Candida*, and *Aspergillus*, *Bacillus Calmette*‐*Guérin* (BCG), and *Mycobacterium tuberculosis* are also relevant pathogens in developing countries, where tuberculosis is endemic and/or BCG vaccine is routinely given, as in Brazil [3, 4, 5, 6].

The five protein components of the NADPH oxidase are encoded by five genes. Thus, a genetic defect in any of the genes disrupts the corresponding protein and impairs the overall function of the complex, leading to the main forms of CGD: the X‐linked recessive form (X‐CGD), caused by defects in *CYBB* gene (gp91^phox^ protein), and the autosomal recessive forms (AR‐CGD), caused by defects in the following genes: *NCF1* (p47^phox^), *NCF2* (p67^phox^), and *NCF4* (p40^phox^), and *CYBA* (p22^phox^) [7, 8].

Diagnosis of CGD is based on the identification of clinical presentations along with assessment of NADPH oxidase activity through measurement of ROS production, followed by genetic testing, which is used to confirm the exact affected gene. The nitroblue tetrazolium (NBT) and dihydrorhodamine 123 (DHR) tests are widely used to diagnose the disease. However, these tests do not determine the specific genetic form of CGD and may yield false‐positive results related to other conditions where NADPH oxidase activity is also impaired. In this context, genetic testing becomes essential not only for genetic counseling and confirmation of the specific type of CGD but also for the identification and referral of individuals for hematopoietic stem cell transplantation (HSCT), the most effective curative therapy for patients with CGD [1, 5, 9, 10]. In particular, diagnosing CGD1 (involving the *NCF1* gene) can be challenging when only based on traditional sequencing methods because *NCF1* has two nonfunctional pseudogenes (Ψ*NCF1*), which share 99% sequence homology with the functional gene. One of the few distinguishing features among them is a GT deletion (ΔGT) at the beginning of exon 2, naturally present in Ψ*NCF1*, which can also arise in functional *NCF1* alleles, resulting in the frameshift variant p.Tyr26HisfsTer [11].

Despite numerous advances in recent decades, the diagnostic process for IEIs, including CGD, remains challenging. Many patients still undergo a diagnostic odyssey without reaching a definitive conclusion. This has a significant impact on their quality of life, delaying both preventive care and potential treatments [12, 13].

Given the clinical relevance of CGD, the scarcity of studies correlating clinical and genetic aspects of the disease, and the need to establish efficient genetic and molecular diagnostic protocols in a genetically diverse population, we characterized a cohort of Brazilian patients through clinical and genomic analyses. We identified the causative genes and variants, performed structural molecular analyses, and described for the first time the p.Cys257Ser variant in the *CYBB* gene and proposed the hypothesis of genotype resulting from recombination events involving *NCF1* and its pseudogenes.

## 2 Materials and Methods

This study was approved by the Research Ethics Committee of the *Hospital de Clínicas de Porto Alegre* (CEP‐HCPA FIPE 2022‐0206). Written informed consent was obtained from all participants or their legal guardians. Six male patients from Southern Brazil with clinical suspicion of CGD had revised their clinical information from medical records and blood samples collected in EDTA vacuum tubes; genomic DNA was extracted using the Easy‐DNA Purification Kit (Thermo Fisher) and quantified using the NanoDrop 1000 spectrophotometer (Thermo Fisher). A custom gene panel was designed using the Ion AmpliSeq Designer software (Thermo Fisher) and included the following genes: *CYBB*, *CYBA*, *NCF1*, *NCF2*, and *NCF4* (Table 1). Sequencing was performed on the Ion Torrent PGM platform (Thermo Fisher) with a minimum coverage of 150×. Data processing was conducted using Torrent Suite v5.0.5, and the GRCh37.p13 assembly was used as the reference genome. Whole genome sequencing was performed on the MGI PCR‐free platform and processed with the Sentieon Germline Pipeline v1.0, using the GRCh38 assembly as the reference genome, with a mean coverage of 30×. The gene‐scan method was employed according to Dekker et al. [11] in order to identify the ΔGT and distinguish the *NCF1* gene from its pseudogenes (Ψ*NCF1*). The fragments were separated by capillary electrophoresis using the ABI 3500 Genetic Analyzer (Applied Biosystems), with the GS500(‐250)LIZ size standard. Electropherogram analysis and peak height identification for *NCF1* and Ψ*NCF1* were performed using Microsatellite Analysis software on the Thermo Fisher Cloud platform. Variant analysis was performed using the following tools: Variant Effect Predictor [14], Ion Reporter (Thermo Fisher), and Integrative Genomics Viewer [15]. Variant classification followed the criteria of the American College of Medical Genetics and Genomics (ACMG) [16] and the Clinical Genome Resource [17].

**TABLE 1:** Genes involved with chronic granulomatous disease.

Cell location	Gene	Protein	Omim	Cytogenetic location	Inheritance	Type of disease
Cell/phagosome membrane	*CYBB*	gp91^phox^	300481	Xp21.1‐p11.4	X‐linked	X‐CGD
	*CYBA*	p22^phox^	608508	16q24.2	Autosomal recessive	CGD4
Cytoplasm	*NCF1*	p47^phox^	608512	7q11.23		CGD1
	*NCF2*	p67^phox^	608515	1q25.3		CGD2
	*NCF4*	p40^phox^	601488	22q12.3		CGD3

The amino acid sequences of gp91^phox^ from 10 different species were retrieved from the Ensembl database [18] to perform a multiple sequence alignment, which was carried out using the MUSCLE software [19]. The three‐dimensional structure of the NADPH oxidase was obtained from the Research Collaboratory for Structural Bioinformatics Protein Data Bank (RCSB PDB) under the identifier 8WEJ [20]. Structural modeling and image generation of the p.Cys257Arg and p.Cys257Ser variants were conducted using PyMOL software [21]. Both wild‐type and mutant NADPH oxidase structures were submitted to structural repair using FoldX v5.0 [22]. Gibbs free energy (ΔG) values were calculated for the overall structure and for interchain interactions in the wild‐type (ΔGwt) and mutant (ΔGmut) NADPH oxidase complexes; differences in ΔΔG (ΔGmut − ΔGwt) greater than +1.6 kcal/mol were considered indicative of significant alterations to the complex [23, 24].

## 3 Results

**FIGURE 1:**
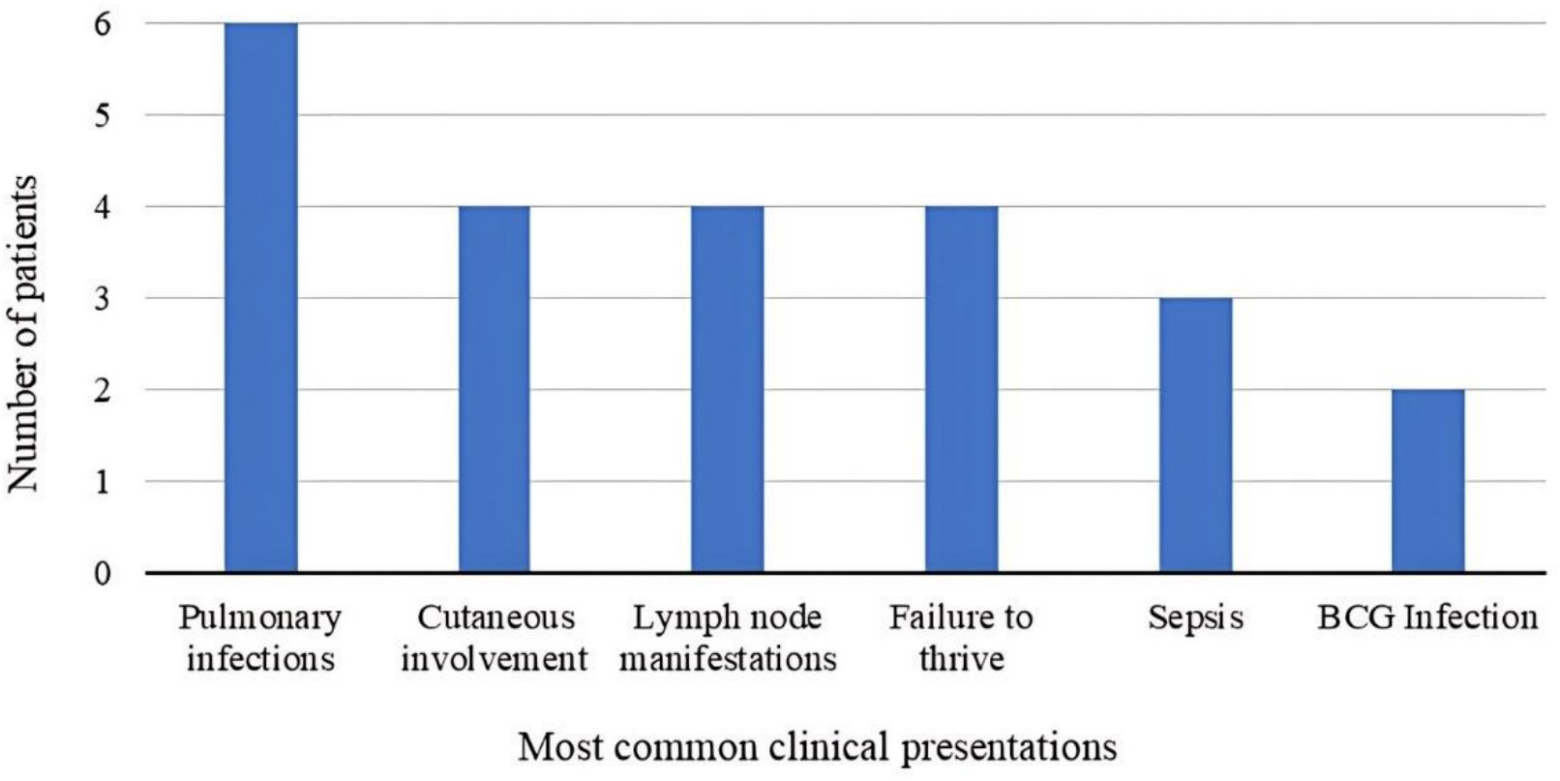
Most common clinical presentations observed in patients P1–P6.

Six male patients were analyzed. Clinical and biochemical information are shown in Table 2, and the genetic information is shown in Table 3. The mean age at symptom onset was approximately 18 months (6 days—6 years). The mean age at the clinical diagnosis was 7.5 years (*n* = 5; 3 months—25 years). The mean age at the genetic diagnosis was 13.1 years (*n* = 6; 1–39 years). The most common manifestations involved recurrent infections mainly on the lungs, observed in all patients, including bronchopneumonia, bronchiolitis, pneumocystis pneumonia, and recurrent pneumonia, and on the skin, observed in 85% of the patients (*n* = 4), including allergic dermatosis, seborrheic dermatitis, infectious pyoderma, and other cutaneous infections (Figure 1). The lymph nodes were also commonly affected, with manifestations occurring in 85% of the patients (*n* = 4), including lymphadenitis, lymphadenomegaly, and recurrent lymphadenopathy.

**TABLE 2:** Clinical and biochemical information of patients P1–P6.

Patients (*n* = 6)	P1	P2	P3	P4	P5	P6
Sex	Male	Male	Male	Male	Male	Male
CGD type	X‐CGD	X‐CGD	X‐CGD	X‐CGD	DCG1	DGC1
Age of onset	6y	4m	6d	2m	8m	1y 9m
Age of clinical diagnosis	25y	4y	6m	3m	NA	8y
Age of genetic diagnosis	39y	11y	5y	1y 1m	6y	17y
Consanguinity	NA	No	No	NA	Yes	Yes
Frequent infection	Yes	Yes	Yes	Yes	Yes	Yes
First clinical manifestation	Cutaneous lesions	Recurrent pneumonia	Infectious pyoderma	Bronchiolitis	Sepsis	Recurrent pneumonia
Granuloma formation	Yes	NA	Yes	Yes	Yes	NA
Other clinical manifestations during follow‐up	BCG infection Bronchopneumonia Chronic lymphadenitis Disseminated fungi infection Lung lesions Lymphadenomegaly Recurrent allergic dermatosis Sepsis	Auricular abscess Bronchopneumonia Failure to thrive Lymphadenomegaly Pneumocystis pneumonia Sepsis	Aspergillosis Disseminated BCG infection Bronchiolitis Failure to thrive Hemorrhagic cystitis Lymphadenomegaly Osteomyelitis Recurrent pneumonia Recurrent cutaneous infections and suppurative lesions Sepsis	Anemia Diarrhea Chronic lymphadenitis Lymphadenomegaly Low Birth Weight Recurrent lymphadenopathy	Chronic rhinitis Failure to thrive Recurrent cutaneous infections and lesions Respiratory infection Seborrheic dermatitis	Bronchopneumonia Failure to thrive Recurrent aphthous ulcers Recurrent otitis Allergic dermatosis
Detected pathogens	*Inonotus tropicalis* *Pseudomonas aeruginosa*	*Pneumocystis jirovecii*	*Aspergillus fumigatus* *Cytomegalovirus* *Klebsiella pneumoniae* *P. aeruginosa*	NA	NA	*Cytomegalovirus*
IgM (RV)	118 mg/dL (50–320)	84 mg/dL (19–146)	106 mg/dL (3m–1y: 17–150)	NA	NA	NA
IgG (RV)	2569 mg/d^a^ (700–1600)	741 mg/dL (453–916)	1178 mg/dL^a^ (30d–1y: 203–948)	NA	1281 mg/dL (2–80y: 540–1822)	1318 mg/dL (2–80y: 540–1822)
IgA (RV)	888 mg/dL^a^ (100–490)	166 mg/dL^a^ (20–100)	264 mg/dL^a^ (3m–1y: 8–91)	NA	198 mg/dL (1–11y: 21–291)	664 mg/dL^a^ (34–305)
IgE (RV)	286 uL/mL^a^ (≤ 100)	25 UI/mL^b^ (10–15y: < 200)	NA	NA	1483 UI/mL^a^ (6–9y: < 90)	NA
C‐reactive protein (RV)	81.60 mg/L^a^ (≤ 10.0)	0.5 mg/dL (< 5.0)	118.7 mg/dL^a^ (< 5.0)	NA	2.1 mg/dL (< 5.0)	10.8 mg/dL^a^ (< 5.0)
CD3+ (RV)	920.4 uL (19–44y: 844–1943)	3615.6 uL (2–6y: 1515–3701)	5071 uL^a^ (6–12m: 2153–5004)	NA	NA	1333 uL (1045–2760)
CD3+/CD4+ (RV)	542.8 uL (19–44y: 476–1136)	2449.7 uL^a^ (2–6y: 618–1348)	3538 uL^a^ (6–12m: 1360–3066)	NA	NA	61 uL (550–1680)
CD3+/CD8+ (RV)	220.7 uL^b^ (19–44y: 248–724)	828.7 uL (2–6y: 453–1700)	1250 uL (6–12m: 560–1803)	NA	NA	599 uL (285–1175)
CD19+ (RV)	88.5 uL^b^ (19–44y: 138–544)	926.1 uL (2–6y: 931–1283)	1453 uL (6–12m: 811–1792)	NA	NA	427 uL (160–600)
CD3‐/CD16+56+ (RV)	171.1 uL (19–44y: 134–545)	754.4 uL^a^ (2–6y: 135–601)	726 uL (6–12m: 164–801)	NA	NA	122 uL (110–910)
DHR‐Assay Ref. 1	NS: 1.80 (C:0.70) WS: 38.50 (C:95.30)	NS: 0.20 (C:0.30) WS: 49.30 (C:84.30)	NS: 0.10 (C:0.40) WS: 0.10 (C:99.40)	NS: 0.10 (C:0.80) WS: 0.30 (C:99.30)	NA	NS: 0.10 (C:0.20) WS: 50.70 (C:89.70)
NBT‐Assay Ref. 2	NS: 1% WS: 5%	NS: 1% WS: 2%	NA	NA	NA	NS: 2% WS: 3%
HSCT	NA	NA	BMT	NA	NA	BMT
Current age	39y	11y	5y	1y10m	6y	17y

*Note:* Ref. 1: NS < 5%, WS > 80%; Ref. 2: NS: 0%–28%, WS: 61%–100%.

Abbreviations: BMT, bone marrow transplantation; C, control values obtained from clinical laboratory tests; d, days; DHR, dihydrorhodamine‐123; HSCT, hematopoietic stem cell transplantation; m, months; NA, not analyzed; NBT, nitroblue tetrazolium; NS, no stimulation; RV, reference values obtained from clinical laboratory tests; WS, with stimulation; y, years.

^a^ Values higher than RV.

^b^ Values lower than RV.

**TABLE 3:** Genetic information of patients P1–P6.

Patients (*n* = 6)	P1	P2	P3	P4	P5	P6
Affected gene	*CYBB*	*CYBB*	*CYBB*	*CYBB*	*NCF1*	*NCF1*
Protein	gp91^phox^	gp91^phox^	gp91^phox^	gp91^phox^	p47^phox^	p47^phox^
Variant type	Missense	Missense	Nonsense	Nonsense	Frameshift	Frameshift
Nucleotide (c.)	c.769T>A	c.769T>C	c.469C>T	c.1449G>A	c.75_76delGT	c.75_76delGT
Amino acid (p.)	p.Cys257Ser	p.Cys257Arg	p.Arg157Ter	p.Trp483Ter	p.Tyr26HisfsTer	p.Tyr26HisfsTer
Location	Exon 7	Exon 7	Exon 5	Exon 11	Exon 2	Exon 2
Zygosis	Hemizygous	Hemizygous	Hemizygous	Hemizygous	Heterozygous	Homozygous
ACMG criteria	PM1 PM2 PM3 PP3	PM1 PM2 PM3 PP3	PVS1 PM3 PP3	PVS1 PM3 PP3	PVS1 PM1 PM2 PM4 PP3	PVS1 PM1 PM2 PM4 PP3
Classification	Likely pathogenic	Likely pathogenic	Pathogenic	Pathogenic	Pathogenic	Pathogenic
*NCF1*/Ψ*NCF1* peak heights	1787/4070	4445/9735	13,947/29,494	4252/8980	Abs./12,569	2222/15,754
*NCF1*/Ψ*NCF1* ratio	2:4 (0.44)	2:4 (0.46)	2:4 (0.47)	2:4 (0.47)	0:6 (0)	1:5 (0.14)

Abbreviation: ACMG, American College of Medical Genetics and Genomics.

**FIGURE 2:**
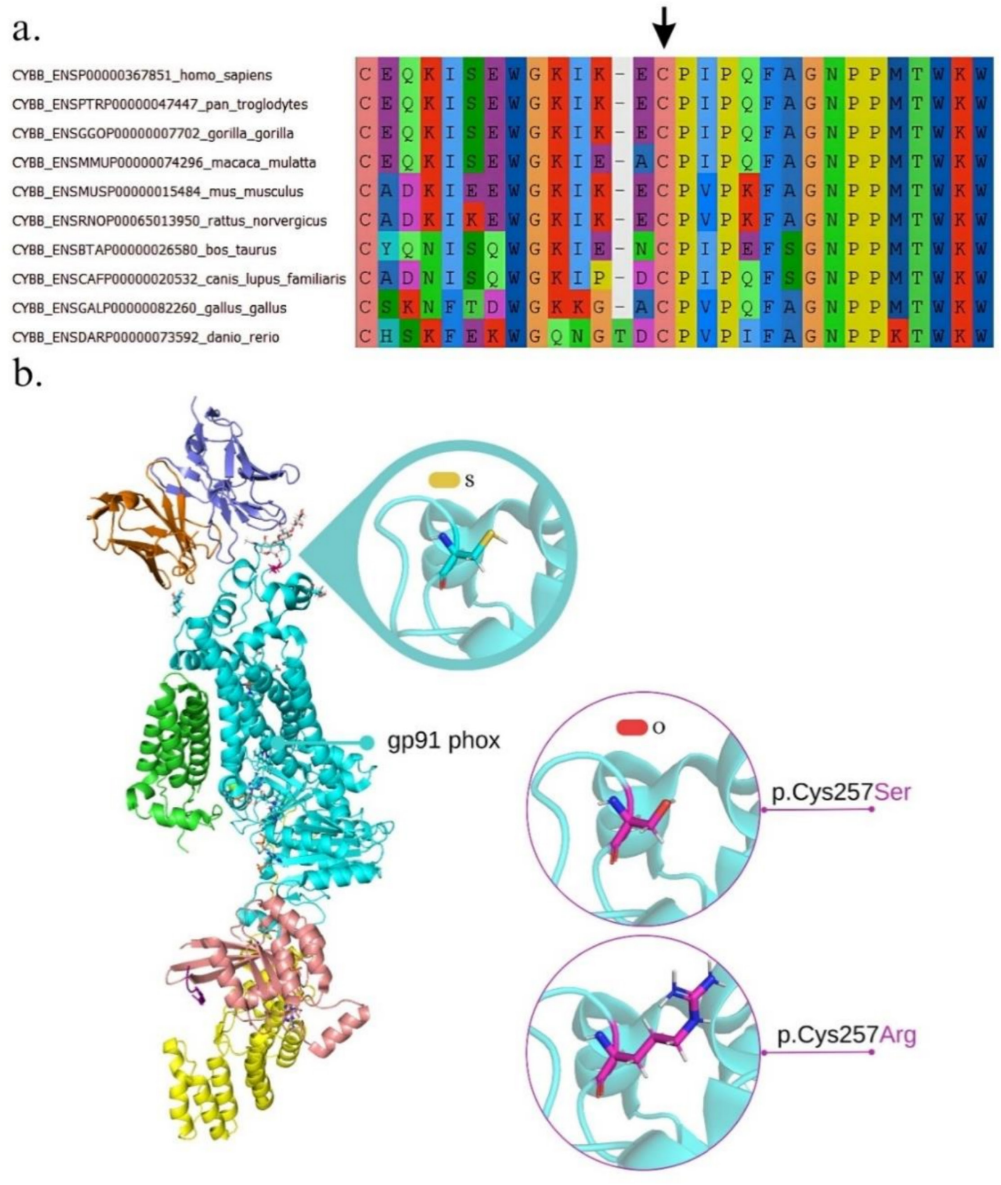
Three‐dimensional modeling of NADPH oxidase and multiple sequence alignment for a cysteine residue in gp91^phox^ across 10 different species. (a) Multiple sequence alignment of the gp91^phox^ amino acid sequence across 10 different species using MUSCLE v3.8, revealing the high conservation of the Cys257 residue (indicated by the arrow). (b) Structural representation of NADPH oxidase, showing the wild‐type cysteine residue in the position 257 in gp91^phox^ subunit and the two missense variants identified in this study (highlighted in magenta).

Four patients were identified with alterations in the *CYBB* gene with four different variants, one of which (p.Cys257Ser) is novel in the literature (P1; Table 3). Multiple sequence alignment across 10 different species demonstrated that the cysteine residue at position 257, where both missense variants in gp91^phox^ occur (p.Cys257Ser and p.Cys257Arg), is highly conserved (Figure 2a). The amino acid substitutions caused by these variants in gp91^phox^ are illustrated in Figure 2b; structural analyses of the NADPH complex for the missense variants revealed significant changes in binding affinity energy (ΔΔG) between chains B (gp91^phox^) and E (a small GTPase) in the p.Cys257Ser variant, as well as in the overall structural stability (ΔΔG_total_) of both variants (Table 4).

**TABLE 4:** ΔΔG (kcal/mol) values of the two missense variants identified in *CYBB* gene found in patients P1 and P2.

Interaction energy		(ΔΔG = ΔGmut − ΔGwt)	
Chains	Wild type	p.Cys257Ser	p.Cys257Arg
AB	−35.38	0	0
BC	0	0	0
BD	−30.82	0.55	0.64
BE	−16.58	2.43	0.15
BH	−2.47	1.23	0.03
BL	−0.49	−0.75	−0.79

Gibbs free energy		(ΔΔG_total_ = ΔGmut − ΔGwt)
Wild type	−61.41	—
p.Cys257Ser	−54.75	6.66
p.Cys257Arg	−55.18	6.23

*Note:* Values greater than +1.6 kcal/mol suggest affinity loss between the chains of the NADPH oxidase and loss of stability in the overall structure.

**FIGURE 3:**
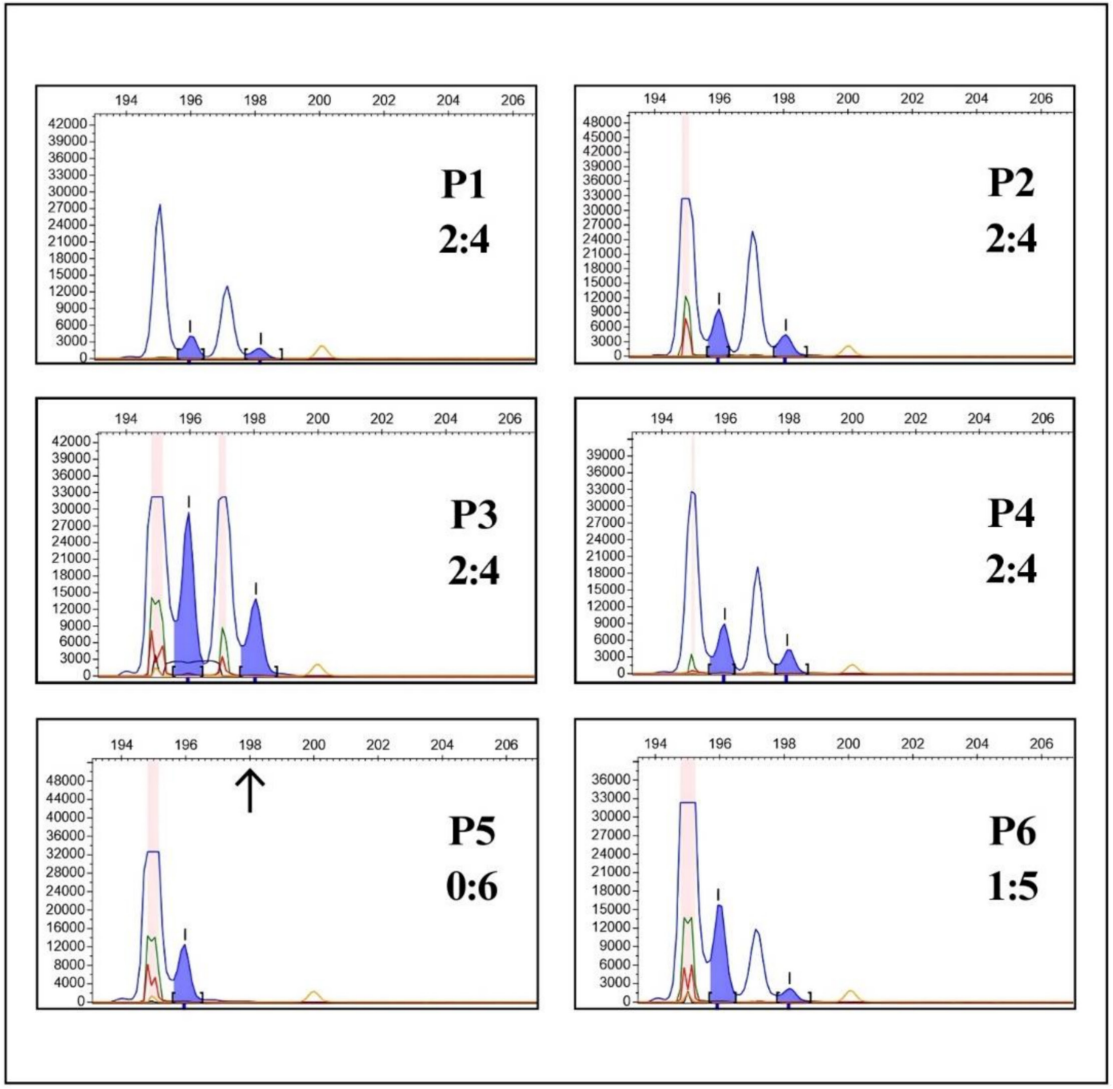
Fragment analysis by the gene‐scan technique of patients P1–P6. The blue peaks on the left correspond to Ψ*NCF1* (196 bp), and the blue peaks on the right correspond to *NCF1* (198 bp). P1–P4 had ratios close to 0.50, reflecting a 2:4 proportion of *NCF1*/Ψ*NCF1*, considered normal values in individuals without ΔGT in the functional *NCF1* gene; P5 had a ratio of zero, indicating homozygosity for the ΔGT in functional *NCF1* gene (arrow indicates absence of functional *NCF1* alleles). P6 had a ratio of 0.14 and a 1:5 proportion, consistent with a heterozygous ΔGT in one functional *NCF1* allele.

**FIGURE 4:**
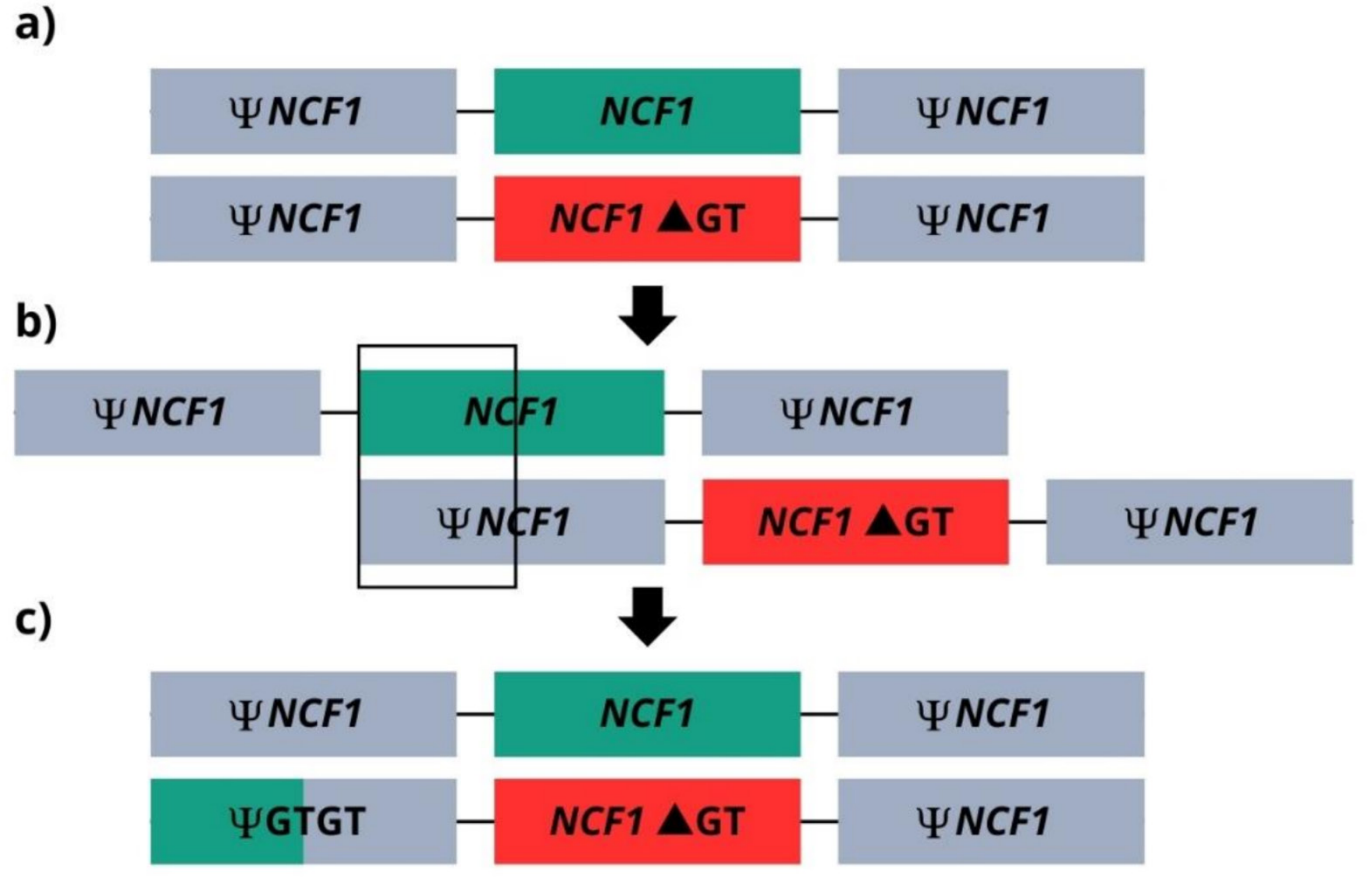
A possible recombination event between *NCF1* and Ψ*NCF1* as a hypothesis for the observed heterozygous ΔGT profile in P6. (a) Both parents of P5 and P6 are likely heterozygous for the ΔGT, as supported by the homozygous genotype observed in P5. (b) An unequal recombination event, previously described by Hayrapetyan et al. [25], may have occurred in one parent, in which *NCF1* and Ψ*NCF1* exchanged their first segment, leading to the insertion of the ΔGT into functional *NCF1* alleles and, simultaneously, the insertion of the normal GTGT sequence into Ψ*NCF1*. (c) A possible explanation for the genotype of P6 is the inheritance of a Ψ*NCF1* carrying the GTGT sequence from one parent, which could confound the analyses by being interpreted as a functional *NCF1* sequence. Adapted from [25].

The gene‐scan results are shown in Figure 3. The analysis revealed the p.Tyr26HisfsTer (ΔGT) variant in *NCF1* in homozygous form in patient P5 and in heterozygous form in patient P6, possibly due to a Ψ*NCF1* lacking the ΔGT, which may have arisen from unequal recombination event (Figure 4). After whole genome sequencing analysis performed in P6, no additional variants in CGD‐associated genes were identified.

## 4 Discussion

This is the first genetic study conducted in a cohort with CGD patients from Southern Brazil. Previous studies in Brazil have been mostly concentrated in the Southeast region, which harbors one of the most admixed populations worldwide. In contrast, the population of Southern Brazil is characterized by a predominantly European ancestry, accounting for approximately 80% of its genetic background [26, 27, 28, 29, 30, 31, 32, 33, 34, 35, 36].

In general, lymphadenitis is the first clinical manifestation observed in patients with CGD. However, in the present study, the first clinical manifestations involved pulmonary infections in 50% of the patients (*n* = 3), cutaneous infections in 33% (*n* = 2), and sepsis in 16% (*n* = 1). Pneumonia is the most common clinical manifestation observed in CGD, affecting 70%–80% of affected patients, and all the patients analyzed were affected by recurrent pneumonia. In this cohort, the organs most frequently affected by infections were the lungs, skin, and lymph nodes, consistent with the literature [8, 10, 37, 38, 39, 40, 41, 42]. Granuloma formation is a typical feature of CGD, which occurs due to excessive inflammatory response, and it was registered in 66.67% (*n* = 4) of the analyzed patients. Genetic variation among innate immunity molecules may contribute to differences in the individual inflammatory response and their severity, modifying the risk of developing inflammatory complications in patients with CGD [6].

Variants in *CYBB* account for more than 60% of all reported CGD cases, representing the leading cause of the disease and predominantly affecting males. Variants in *NCF1* contribute to approximately 20% of cases and represent the most frequent cause of AR‐CGD; variants in *CYBA* and *NCF2* are responsible for about 14% of cases [2]. Among the patients analyzed, 66.67% (*n* = 4) were diagnosed with X‐CGD caused by *CYBB* variants, and 22.23% (*n* = 2) with AR‐CGD due to the ΔGT in *NCF1*. Only 50% of these patients (*n* = 3) had previously received a clinical diagnosis based on biochemical testing, but without a definitive classification of the CGD subtype.

The most severe cases of CGD are associated with extremely low or absent ROS levels, usually resulting from nonsense *CYBB* variants that cause complete loss of gp91^phox^ function and tend to present earlier in life. Such was the case of patient P3, who developed symptoms at 6 days of life, representing the most severe phenotype among those analyzed. In contrast, AR‐CGD cases in general, as well as some X‐linked cases caused by missense variants affecting amino acids 1–309 (except residue 222) of gp91^phox^, are associated with better survival. These patients usually present with milder to moderate phenotypes due to residual ROS production, which can cause a delayed onset of symptoms as well as a delay in definitive diagnosis, contributing to the overall low diagnosis rate [1, 7, 8, 12, 43].

Most patients with CGD are diagnosed before the age of five. However, in the present cohort, only one patient (P4) received both clinical and genetic diagnoses within this age range. Late diagnosis is common among Brazilian patients, with reported ages at diagnosis ranging from 8 to 19 years [28, 32, 35, 36, 38, 40, 44, 45].

Two patients (P1 and P3) had infections resulting from BCG vaccination, P3 with the disseminated form. Complications related to BCG vaccination are commonly observed in patients with CGD, due to their inability to eliminate the attenuated *Bacillus*. Data from Latin America and Asia show that such complications occur in 11%–58% of patients with CGD and may represent the first clinical manifestation of the disease. Although rare, disseminated BCG infection is associated with a mortality rate of 60%–80%. In Brazil, the BCG vaccine is routinely administered to newborns and children before 5 years of age because of tuberculosis endemism in the country. Therefore, it is crucial to contraindicate the BCG vaccination for patients with CGD [3, 30, 40, 41, 44].

Genetic testing plays a key role in the efficient and early determination of the specific type of CGD, contributing to appropriate disease management and genetic counseling, as well as supporting the selection and referral of patients for HSCT [5, 7, 10, 39, 46].

Structural alterations in gp91^phox^ can affect the electron transfer site from NADPH to O_2_ in extra‐ or intracellular compartments, thereby determining ROS production [47]. Two patients diagnosed with X‐CGD had missense variants in the *CYBB* gene, in which the p.Cys257Ser variant identified in patient P1 is a novel finding in the literature and was classified as likely pathogenic. This patient was clinically and genetically diagnosed at the ages of 25 and 39, respectively. Barkai et al. [48] also reported CGD patients with *CYBB* missense variants in Israel with delayed diagnosis, with a mean diagnostic age of 57.3 years. In patient P2, the likely pathogenic variant p.Cys257Arg was identified, which was previously described in Japanese and Chinese patients. Although BCG infections were identified in the Japanese cohort, no genotype correlation was observed, and such infections were not reported in P2 [49, 50, 51].

The Cys257 residue in gp91^phox^ affected by *CYBB* missense variants is located in loop E, the largest among the loops of gp91^phox^ (loops A and C), a region of structural and functional relevance. Loop E also contains an N‐linked glycosylation site essential for protein folding, stability, and function. In addition, this loop stabilizes and correctly positions an external heme group of the gp91^phox^, which is crucial for electron transfer and ROS generation. Furthermore, residues Cys257 and Cys244 form a disulfide bond within gp91^phox^, which plays a fundamental role in maintaining tertiary and quaternary conformation of proteins. Both variants involving the Cys257 residue were previously predicted by Noreng et al. [52] to disrupt the structure of loop E. In addition, this loop is highly conserved among mammals as well as this cysteine residue, which is conserved across 10 species, emphasizing its biological and evolutionary significance [20, 46, 53, 54, 55, 56].

The ΔΔG_total_ values for both variants observed in patients P1 and P2 revealed a stability loss in the overall structure of the NADPH oxidase, suggesting a significant functional impact; ΔΔG values greater than 3 kcal/mol indicate a high destabilization in the protein structure. In addition, ΔΔG values of interchain affinity energies in B and E chains of p.Cys257Ser were found to be significant, suggesting an impact on the interaction between gp91^phox^ and a small GTPase (*RAC1/2*), which may affect the activation of the NADPH oxidase [20, 23, 47, 57, 58].

The nonsense variant p.Arg157Ter in *CYBB*, identified in patient P3, was classified as likely pathogenic and is reported in more than 60 patients across Latin America, North America, Asia, and Europe [41, 59, 60, 61, 62, 63, 64, 65, 66]. This variant occurs within a CpG island involved in transcriptional regulation through cytosine demethylation, which is considered a mutational hotspot in *CYBB*. Moreover, it was previously reported in Brazil, where the absence of gp91^phox^ expression was demonstrated in a patient [26, 31].

In patient P4, the pathogenic variant p.Trp483Ter in *CYBB* was identified. This variant has been reported in studies from India and Denmark, with the respective patients receiving a diagnosis before the age of 5, as was the case with P4 [64, 67]. The Indian patient reported by Rawat et al. [64] died within the first year of life, whereas the Danish patient presented with chronic urticaria and cerebral abscesses. This variant is predicted to undergo nonsense‐mediated mRNA decay (NMD), a mechanism that targets transcripts harboring a premature termination codon located more than 50 nucleotides upstream of the last exon–exon junction, thereby preventing accumulation of nonfunctional transcripts and aberrant proteins, leading to the absence of gene products [68]. In addition, alterations occurring beyond residue 310 of gp91^phox^ impair the binding domains for FAD and NADPH, which are crucial for ROS production [7, 46].

The p.Tyr26HisfsTer (ΔGT) variant in *NCF1*, identified in patients P5 and P6, accounts for more than 90% of all AR‐CGD cases and leads to loss of function of p47^phox^. Due to the high homology between *NCF1* and its pseudogenes, several hotspots for recombination events exist, which can occur unequally and insert the ΔGT in *NCF1*, which is estimated to occur in 1 out of every 250 individuals. Patients P5 and P6 are siblings from consanguineous parents. P5 is homozygous for ΔGT, whereas P6 was identified as heterozygous by gene‐scan, gene panel, and whole genome analysis. Despite presenting a clinical phenotype of CGD, no additional variants were detected in *NCF1* or in the other CGD‐associated genes in P6. A possible explanation for this heterozygous profile is the presence of a Ψ*NCF1* carrying the GTGT sequence instead of the ΔGT, as previously described by Heyworth et al. [69] and Hayrapetyan et al. [25], which may have arisen through unequal recombination with *NCF1*. Considering that the parents are consanguineous and likely heterozygous for ΔGT, as indicated by the homozygosity observed in P5, it is plausible that a recombination event occurred during gametogenesis, leading to the observed genotype discrepancy between the siblings (Figure 4). Moreover, the high degree of homology between *NCF1* and its pseudogenes complicates sequencing analysis, frequently resulting in alignment errors, misreads, and incorrect variant calls. Furthermore, unequal recombination events can cause deletion of entire gene segments, which are also difficult to detect using conventional sequencing methods or even the gene scan [2, 11, 70, 71, 72].

## 5 Conclusions

CGD belongs to a group of rare and underdiagnosed diseases, highlighting the importance of characterizing clinical presentations and establishing a genetic diagnosis through genotype–phenotype correlations. Beyond guiding appropriate patient management and genetic counseling, genetic diagnosis contributes to the referral of patients for HSCT and enables the contraindication of BCG vaccination, thereby preventing adverse and potentially life‐threatening reactions. In the present study, it was possible to document both the clinical and genetic heterogeneity of Brazilian patients with CGD, as well as to identify and characterize a novel variant causing the disease.

## Author Contributions

**Leonardo Martinello da Rosa:** data curation, formal analysis, investigation, methodology, project administration, software, validation, visualization, writing – original draft, writing – review and editing. **Martha Braun da Rosa:** formal analysis, investigation, methodology, software, visualization, writing – review and editing. **Mariana de Sampaio Leite Jobim Wilson:** conceptualization, data curation, investigation, methodology, resources, validation, writing – review and editing. **Ida Vanessa Doederlein Schwartz:** conceptualization, data curation, funding acquisition, methodology, project administration, resources, supervision, writing – review and editing. **Fernanda Sperb‐Ludwig:** conceptualization, data curation, formal analysis, funding acquisition, investigation, methodology, project administration, resources, software, supervision, validation, visualization, writing – original draft, writing – review and editing.

## Funding

This work was supported by Conselho Nacional de Desenvolvimento Científico e Tecnológico; Fundação de Amparo à Pesquisa do Estado do Rio Grande do Sul (FAPERGS 24/2551‐0000591‐9), and Fundo de Incentivo à Pesquisa e Eventos do Hospital de Clínicas de Porto Alegre (2022‐0206).

## Ethics Statement

This study was approved by the Research Ethics Committee of *Hospital de Clínicas de Porto Alegre* under protocol number 2022‐0206, approved on December 28, 2022, in conformity with the principles of the Declaration of Helsinki.

## Consent

Written informed consent was obtained from all participants or their legal guardians prior to inclusion in the present study.

## Conflicts of Interest

The authors declare no conflicts of interest.

## Data Availability

The datasets generated and analyzed during the current study include clinical information and genetic sequencing files. Due to the sensitive nature of these data and patient privacy concerns, they are not publicly available. However, data may be made available upon reasonable request, which will be assessed by the authors in consultation with the corresponding institutional ethics committee.
